# Methylation divergence of invasive *Ciona* ascidians: Significant population structure and local environmental influence

**DOI:** 10.1002/ece3.4504

**Published:** 2018-09-05

**Authors:** Ping Ni, Shiguo Li, Yaping Lin, Wei Xiong, Xuena Huang, Aibin Zhan

**Affiliations:** ^1^ Research Center for Eco‐Environmental Sciences Chinese Academy of Sciences Beijing China; ^2^ University of Chinese Academy of Sciences Chinese Academy of Sciences Beijing China

**Keywords:** biological invasion, DNA methylation, methylation divergence, methylation‐sensitive amplified polymorphism, tunicate

## Abstract

The geographical expansion of invasive species usually leads to temporary and/or permanent changes at multiple levels (genetics, epigenetics, gene expression, etc.) to acclimatize to abiotic and/or biotic stresses in novel environments. Epigenetic variation such as DNA methylation is often involved in response to diverse local environments, thus representing one crucial mechanism to promote invasion success. However, evidence is scant on the potential role of DNA methylation variation in rapid environmental response and invasion success during biological invasions. In particular, DNA methylation patterns and possible contributions of varied environmental factors to methylation differentiation have been largely unknown in many invaders, especially for invasive species in marine systems where extremely complex interactions exist between species and surrounding environments. Using the methylation‐sensitive amplification polymorphism (MSAP) technique, here we investigated population methylation structure at the genome level in two highly invasive model ascidians, *Ciona robusta* and *C. intestinalis,* collected from habitats with varied environmental factors such as temperature and salinity. We found high intrapopulation methylation diversity and significant population methylation differentiation in both species. Multiple analyses, such as variation partitioning analysis, showed that both genetic variation and environmental factors contributed to the observed DNA methylation variation. Further analyses found that 24 and 20 subepiloci were associated with temperature and/or salinity in *C. robusta* and *C. intestinalis*, respectively. All these results clearly showed significant methylation divergence among populations of both invasive ascidians, and varied local environmental factors, as well as genetic variation, were responsible for the observed DNA methylation patterns. The consistent findings in both species here suggest that DNA methylation, coupled with genetic variation, may facilitate local environmental adaptation during biological invasions, and DNA methylation variation molded by local environments may contribute to invasion success.

## 1 INTRODUCTION

Biological invasions provide promising “natural experiments” to advance our understanding of rapid environmental adaptation over contemporary time scales (Colautti & Lau, 2015; Lee, 2002; Prentis, Wilson, Dormontt, Richardson, & Lowe, 2008; Shine, 2012). Invasive species can rapidly spread over broad geographical scales by natural and/or human‐mediated dispersal and successfully colonize diverse habitats with varied local environmental conditions (Doney et al., 2012; Zhan, Briski, Bock, Ghabooli, & Macisaac, 2015). For marine invaders, introductions over long‐distance appear to be occurring with increased frequency due to intense human activities, such as shipping, aquaculture, and aquarium trades (Carlton, 1996; Lin, Gao, & Zhan, 2015; Zhan et al., 2017). In invaded habitats, invaders are often challenged by dramatically different abiotic and biotic conditions (Darling, Bagley, Roman, Tepolt, & Geller, 2008; Lee, 1999; Paul‐Pont, De Montaudouin, Gonzalez, Soudant, & Baudrimont, 2010; Sorte, Jones, & Miller, 2011; Zhan et al., 2015). Numerous studies suggest that rapid microevolution can occur in response to strong and persistent environmental challenges during biological invasions (Bock et al., 2015; Huang et al., 2017; Lande, 2015; Lin et al., 2017; Pu & Zhan, 2017; Sherman et al., 2016).

Epigenetic modification represent one important rapid mechanism for invasion success, as epigenetic modifications can influence phenotypes by regulating gene expression without changing the underlying DNA sequences (Bossdorf, Richards, & Pigliucci, 2008; Hawes et al., 2018; Rapp & Wendel, 2005). Although epigenetic variation can be predicted from its genotypic contexts or specific genetic polymorphisms elsewhere in the genome, still part of epigenetic variation can be independent from genetic control and is involved in response to environmental challenges as an autonomous system (Richards, 2006, 2008). Various types of epigenetic modifications, including modifications of DNA, histones and other chromosomal proteins, and the generation of extrachromosomal regulatory small RNAs and noncoding RNAs, have been found in many eukaryotes (Richards, 2008). Among these epigenetic modifications, DNA methylation, the addition of a methyl group to a specific base (mostly cytosine for eukaryotes), is one of the most intensively studied for its ubiquity in eukaryotes and essential biological functions (Jones, 2012; Suzuki & Bird, 2008). DNA methylation is involved in numerous biological processes, such as blocking transcription initiation, controlling transcription elongation, silencing transposon, inactivating X chromosome, and imprinting on genes (Duncan, Gluckman, & Dearden, 2014; Jones, 2012). In addition, studies of DNA methylation have been advanced by the development of new techniques, such as bisulfite conversion‐based methods (e.g., whole genome bisulfite sequencing—WGBS; reduced representation bisulfite sequencing—RRBS), immunoprecipitation‐based methods (e.g., methylated DNA immune‐precipitation sequencing—MeDIP‐seq; methylated DNA‐binding domain sequencing—MBD‐seq), and restriction enzyme‐based methods (e.g., methylation‐sensitive amplified polymorphism—MSAP; Methyl‐seq) (Suzuki & Bird, 2008; Wang et al., 2015). Although bisulfite conversion‐based sequencing techniques have been considered as promising tools for detecting methylation modifications, these methods are limited in studies at the population level (i.e., large‐scale population epigenomics studies), mainly owing to high experimental and computational costs as well as high‐quality reference genomes sequenced and assembled for genomewide analyses (Schulz, Eckstein, & Durka, 2013; Suzuki & Bird, 2008). Instead, the methylation‐sensitive amplification polymorphism (MSAP) technique, which can detect different methylation status at random restriction sites over a genome, is favorable as it allows to evaluate genomic methylation patterns of wild populations and identify associations between environmental conditions, phenotypic traits, and methylation status with low cost (Alonso, Pérez, Bazaga, Medrano, & Herrera, 2016). Thus, MSAP is the most widely used method in ecological epigenetics studies at the population level in a wide range taxa (Schulz et al., 2013).

During the process of local accommodation and adaptation, the most important feature of DNA methylation is its environmental effects (Verhoeven, VonHoldt, & Sork, 2016). Studies based on both common garden experiments and populations collected from the wild have detected correlations between DNA methylation variation and environmental stresses, indicating the environmental influence on DNA methylation divergence. For example, greenhouse experiments have shown that various stresses, such as low nutrients, salt stress, dietary components, and pathogen attack, can induce methylation variation throughout the whole genome (Dowen et al., 2012; Huang et al., 2017; Morán, Marco‐Rius, Megías, Covelo‐Soto, & Pérez‐Figueroa, 2013; Platt, Gugger, Pellegrini, & Sork, 2015; Verhoeven, Jansen, van Dijk, & Biere, 2010). In the wild, DNA methylation differentiation among populations has been frequently observed in different environments (Gugger, Fitz‐Gibbon, Pellegrini, & Sork, 2016; Paun et al., 2010; Wenzel & Piertney, 2014). In addition, maintenance and removal of DNA methylation are enzymatically mediated, thus the state of DNA methylation can change rapidly throughout the whole genome (Law & Jacobsen, 2010). Furthermore, changes in DNA methylation states can often cause important phenotypic consequences, leading to rapid accommodation and even adaptation to different local environments (Gao, Geng, Li, Chen, & Yang, 2010; Lea, Altmann, Alberts, & Tung, 2016; Pu & Zhan, 2017). Therefore, DNA methylation can integrate environmental signals into genomes rapidly and thereby modify gene expression and/or phenotypic variation to adapt to environmental changes/stresses.

DNA methylation variation is expected to be involved in the process of accommodation/adaptation to heterogeneous environments for invasive species (Hawes et al., 2018 and references therein; Pu & Zhan, 2017). Recent studies in invasive species showed that methylation diversity was much greater than genetic diversity in introduced populations of Japanese knotweed *Fallopia* species (Richards, Schrey, & Pigliucci, 2012) and Alligator weed *Alternanthera philoxeroides* (Gao et al., 2010). Liebl, Schrey, Richards, and Martin (2013) inferred that high methylation diversity could compensate for reduced genetic diversity during the invasion process of the house sparrow *Passer domesticus*. Interestingly, introduced populations of bluegrass *Poa annua* tended to have higher levels of methylation diversity than native populations (Chwedorzewska & Bednarek, 2012), and studies of the pygmy mussel (*Xenostrobus securis*) and tubeworm (*Ficopomatus enigmaticus*) found that recently introduced populations seemed to be less methylated in comparison with older introduced populations (Ardura, Zaiko, Morán, Planes, & Garcia‐Vazquez, 2017). Importantly, studies of invasive plants have also detected the association between DNA methylation variation and different habitats (Gao et al., 2010; Richards et al., 2012), and the authors further identified habitat‐related methylated loci (Richards et al., 2012). In order to further analyze possible contributions of local environments to methylation differentiation, studies assessed the degree of methylation differentiation among populations sampled from different environments. Interestingly, the level of methylation differentiation highly varied among invasive species, for example, strong methylation divergence was observed among populations of bluegrass (differentiation = 0.5; Chwedorzewska & Bednarek, 2012) and Japanese knotweed (differentiation = 0.5–0.8; Richards et al., 2012), while low methylation differentiation was found in introduced house sparrow populations in Kenya (differentiation = 0.004; Liebl et al., 2013). Collectively, it still remains largely unexplored on methylation patterns and possible contributions of varied environmental factors to methylation differentiation during range expansions in many invasive species.

Here, we used invasive model ascidians *C. robusta* (=*C. intestinalis* sp. A, Brunetti et al., 2015) and *C. intestinalis* (=*C. intestinalis* sp. B, Brunetti et al., 2015) to investigate population methylation structures and possible responsible environmental factors for observed patterns in diverse habitats. *C. robusta* and *C. intestinalis*, two morphologically similar but genetically distinct species of the *C. intestinalis* complex, have widely invaded temperate and warm‐temperate coastal zones over the past century (Carver, Mallet, & Vercaemer, 2006; Zhan, Macisaac, & Cristescu, 2010; Zhan et al., 2015). Due to ambiguous taxonomy within the genus *Ciona*, the native/invaded ranges and invasion histories of both *C. robusta* and *C. intestinalis* remain uncertain (Zhan et al., 2015 and references therein). *C. robusta*, which is generally considered as an East Asian native, has invaded coasts of Mediterranean Sea and many regions of Atlantic and Pacific coasts, while *C. intestinalis*, likely a native of Europe, has widely colonized northeast Atlantic coasts (Bouchemousse, Bishop, & Viard, 2016; Zhan et al., 2015 and references therein). Life history traits of *Ciona*, such as spawning time and life span, have a close relationship with its environmental conditions, particularly water temperature (Carver et al., 2006 and reference therein). For example, deep water populations in Scandinavia and sub‐Arctic populations can live 2–3 years and reproduce only once a year or less, while populations in the shallow regions of Scandinavian coast or in coastal regions of Atlantic Canada can survive for 12–18 months with two recruitment peaks per year (Carver et al., 2006 and reference therein). The broad geographical distribution, as well as varied environments in their native and/or invaded habitats, reflects the wide range environmental tolerance of both invasive species, especially for temperature and salinity (Dybern, 1967; Therriault & Herborg, 2008; Zhan et al., 2015). Both *C. robusta* and *C. intestinalis* have a short pelagic larval phase (1–5 days) and a sessile adult stage, suggesting that natural dispersal can only occur over relatively limited geographical ranges (Zhan et al., 2015). Therefore, the wide range expansion of both invasive species, particularly at the regional and continental scales, mainly is owing to human‐mediated pathways, which results in sudden shifts of habitats with varied environments. As such, these two ascidians provide good models to study mechanisms of rapid microevolution during biological invasions (Lin et al., 2017; Pu & Zhan, 2017; Zhan et al., 2015). Our previous study revealed significant genetic differentiation among *C. robusta* populations collected globally by using genomewide gene‐associated microsatellites, and genetic signatures and loci under selection were associated with varied local environmental conditions (Lin et al., 2017). Furthermore, when we analyzed the DNA methylation variation of salinity and temperature‐related genes using bisulfite sequencing at the population level, we found significant variation of DNA methylation among populations (Pu & Zhan, 2017). Interestingly, frequencies of several CpG loci were significantly correlated with local environmental factors (Pu & Zhan, 2017). However, Pu and Zhan's (2017) only focused on five genes which were putatively responsible for changes of temperature and salinity, and it remains largely unknown how environmental changes associated with habitat invasions shape methylation divergence among *Ciona* populations in different environments at the genome level.

In this study, we investigated the DNA methylation variation in *C. robusta* and *C. intestinalis* populations using the MSAP technique. We analyzed the DNA methylation patterns for populations of both species collected from different continents with varied local environments. In order to interpret the significant population methylation differentiation among populations, the possible contributions of genetic variation and two crucial environmental factors in marine ecosystems (i.e., temperature and salinity) were further tested.

## 2 MATERIALS AND METHODS

### 2.1 Sample collection

**Figure 1:**
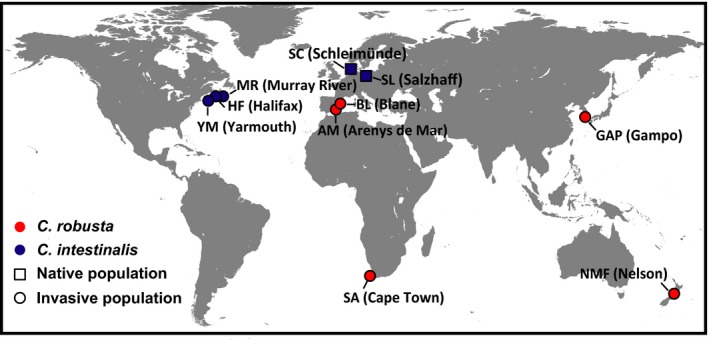
Sampling sites of *Ciona robusta* (red) and *C. intestinalis* (blue)

To cover the substantial environmental gradients in habitats of *C. robusta* and *C. intestinalis*, sampling sites were selected based on our former studies (Zhan et al., 2010, 2012) across four continents (Asia, Africa, Europe, and Oceania) for *C. robusta* and two continents (Europe and North America) for *C. intestinalis* (Table 1; Figure 1). To make it clear for further comparison between genetic and methylation data, populations of both *C. robusta* and *C. intestinalis* were named as our previous studies (Lin et al., 2017; Zhan et al., 2010, 2012). Temperature and salinity, two crucial environmental factors affecting numerous physiological processes in marine invertebrates, have a high degree of variation at the chosen sampling sites in this study (see Lin et al., 2017 for *C. robusta*; Table 2, for *C. intestinalis*). We chose a total of 10 populations, including five populations of *C. robusta* (*N *= 115) and five populations of *C. intestinalis* (*N *= 148; Table 1; Figure 1). For *C. robusta*, all of five collected populations were invasive populations, of which populations AM (Arenys de Mar) and BL (Blanes) established at the end of 19th, while populations SA (Cape Town) and NMF (Nelson) were introduced at mid‐20th (Bouchemousse et al., 2016 and references therein). Population GAP (Gampo) was reported on the coast of Korea at the late 1990s (Seo & Lee, 2009). For *C. intestinalis*, two European populations SL (Salzhaff) and SC (Schleimünde) were considered as putatively native populations, while the other three Canadian populations were invasive populations locally detected after 1997 (Bouchemousse et al., 2016 and references therein). All collected adult specimens were preserved in 100% ethanol at 4°C before analyses. All specimens were identified and confirmed to the species level by using one mitochondrial DNA fragment, cytochrome *c* oxidase subunit 3–NADH dehydrogenase subunit 1 (COX3‐ND1; Zhan et al., 2010).

**Table 1:** Sampling sites and environmental parameters for the two highly invasive ascidians *Ciona robusta* and *C. intestinalis*

Population ID	Region/state and country	Colonized time	Coordinates	*n*	AveT (°C)	MaxT (°C)	MinT (°C)	AveS (‰)	MaxS (‰)	MinS (‰)
*C. robusta*										
AM	Arenys de Mar, Spain	End‐19th	41°33′41″N, 2°34′37″E	29	17.98	25.28	13.29	37.67	38.21	36.87
BL	Blanes, Spain	End‐19th	41°41′12″N, 2°53′22″E	12	17.44	24.38	12.98	37.95	38.32	37.48
SA	Cape town, South Africa	Mid‐20th	33°54′33″S, 18°25′59″E	32	16.03	16.92	15.16	35.18	35.30	34.99
GAP	Gampo, Korea	End‐20th	35°48′26″N, 129°30′13″E	30	17.72	24.31	12.18	33.72	34.48	32.17
NMF	Nelson, New Zealand	Mid‐20th	41°15′29″S, 173°16′42″E	12	13.55	16.37	11.22	34.78	34.92	34.62
*C. intestinalis*										
HF	Halifax, Canada	End‐20th	44°38′48″N, 63°34′8″W	30	8.51	17.98	1.09	31.03	31.54	30.68
MR	Murray River, Canada	Early‐21st	46°0′53″N, 62°36′30″W	30	7.77	18.37	−1.25	29.28	30.64	28.20
YM	Yarmouth, Canada	End‐20th	43°50′06″N, 66°07′22″W	30	7.08	11.48	2.16	31.81	32.20	31.50
SL	Salzhaff, Germany	Putatively native	54°2′22″N, 11°31′36″E	30	9.25	17.44	1.82	15.67	18.52	13.36
SC	Schleimünde, Germany	Putatively native	54°41′25″N, 10°7′15″E	28	9.45	17.94	1.90	14.84	17.67	12.71

Note

S: salinity; T: temperature.

**Table 2:** *p*‐Values for the exact test for the difference of the temperature (above diagonal) and salinity (below diagonal) among sample sites of *Ciona intestinalis* based on a nonparametric test, Mann–Whitney *U* test

	HF	MR	YM	SL	SC
HF		—	—	—	—
MR	a		—	—	—
YM	a	a		—	—
SL	a	a	a		—
SC	a	a	a	—	

a *p* < 0.01; —, not significant.

### 2.2 Methylation‐sensitive amplification polymorphism assay

Total genomic DNA was isolated from approximately 50 mg of siphon tissues following the proteinase K method (Waters, Dijkstra, & Wallis, 2000). The quality and quantity of DNA were measured using Nanodrop 2000 spectrophotometer (Thermo Scientific). Total genomic DNA (300 ng) was digested at 37°C for 3 hr in two parallel reactions using 5 U of *Eco*RI‐HF and 5 U of either *Msp*I or *Hpa*II (New England Biolabs) in a final volume of 10 μl. *Msp*I and *Hpa*II can recognize and cleave the same sequence 5′‐CCGG‐3′, but their susceptibility is different depending on the methylation state of cytosines at restriction sites (Schulz et al., 2013). After digestion, enzymes were heat‐deactivated at 80°C for 10 min. *Eco*RI_ADAPTER (F) 5′‐CTCGTAGACTGCGTACC‐3′/*Eco*RI_ADAPTER (R) 5′‐AATTGGTACGCAGTCTAC‐3′ and *Hpa*II/*Msp*I_ADAPTER (F) 5′‐GACGATGAGTCTAGAA‐3′/*Hpa*II/*Msp*I_ADAPTER (R) 5′‐CGTTCTAGACTCATC‐3′ (Wenzel & Piertney, 2014) were used to prepare *Eco*RI and *Hpa*II/*Msp*I adapters by mixing an equal amount of complementary oligonucleotides. A ligation mixture in a volume of 5 μl contained 5 pmol of *Eco*RI adaptor and 50 pmol of *Hpa*II/*Msp*I adaptor, and 30 U of T4 DNA ligase (New England Biolabs) was added to digestion products and incubated at 37°C for 3 hr, followed by 16°C for 9 hr, and then 65°C for 20 min.

Preselective polymerase chain reaction (PCR) was carried out in a total volume of 20 μl containing 2 μl of the ligation product, 0.4 μM each of *Eco*RI_ADAPTER (F)+A and *Hpa*II/*Msp*I_ADAPTER (F)+T preselective primers, 0.5 U of *Taq* DNA polymerase (Takara), 2 mM of MgCl_2_, and 0.2 mM of each nucleotide. The PCR profile was as follows: 72°C for 2 min followed by 30 cycles of 95°C for 20 s, 56°C for 30 s and 72°C for 1 min, and a final elongation step at 60°C for 2 min.

Based on the number of amplified fragments, five primer pairs (Table 3) were chosen for selective PCRs. Selective PCRs were performed in a total volume of 30 μl using 0.5 μl of preselective PCR product, 0.2 μM of fluorescently labeled (6‐FAM) forward primer, 0.2 μM of reverse primer, 0.5 U of *Taq* DNA polymerase (Takara), 1.25 mM of MgCl_2_ and 0.125 mM of each nucleotide. The PCR profile was as follows: 94°C for 3 min, 13 touchdown cycles of 94°C for 30 s, 65°C for 30 s reduced by 0.7°C per cycle and 72°C for 2 min, 24 cycles of 94°C for 30 s, 56°C for 30 s and 72°C for 2 min, and a final elongation step at 72°C for 2 min. Selective amplification products were separated on an ABI 3730 DNA analyzer (Applied Biosystems) along with GeneScan LIZ 500 size standard (Applied Biosystems). GeneMarker v.2.2.0. (SoftGenetics) was used for fragment scrutiny. GeneMarker panels were created for each primer pair with the fragment range from 150 bp to 500 bp, as fragment size <150 bp may increase the potential impact of size homoplasy (Caballero, Quesada, & Rolán‐Alvarez, 2008). Monomorphic epiloci and singletons (i.e., when only one sample had a deviating status) were excluded from further analyses. Replicated samples (6% of the total) starting from DNA extraction were included in all steps to test the reproducibility of MSAP assay (Bonin et al., 2004). Epiloci with more than two mismatches across the replicated samples were discarded from our datasets. The error rate based on replicated samples was estimated as 5.71% and 5.66% for *C. robusta* and *C. intestinalis*, respectively.

**Table 3:** Selective primer combinations used in methylation‐sensitive amplification polymorphism (MSAP)

*Eco*RI	*Hpa*II/*Msp*I
GACTGCGTACCAATTCACA	GATGAGTCTAGAACGGTTA
GACTGCGTACCAATTCAGA	GATGAGTCTAGAACGGTGTT
GACTGCGTACCAATTCAGA	GATGAGTCTAGAACGGCT
GACTGCGTACCAATTCAGA	GATGAGTCTAGAACGGAT
GACTGCGTACCAATTCATC	GATGAGTCTAGAACGGCA

### 2.3 Data analyses

**Figure 2:**
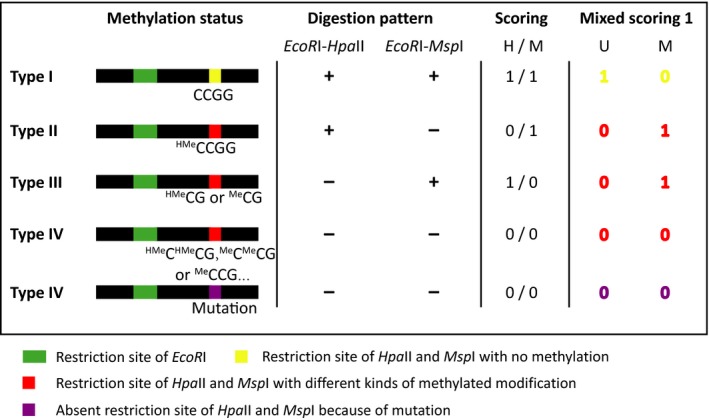
Scoring scheme based on the “Mixed Scoring 1” method

Individuals were scored based on the presence (as “1”) or absence (as “0”) of *Eco*RI‐*Hpa*II and *Eco*RI‐*Msp*I fragments, and four methylation conditions of restriction sites are distinguished as: Type I) fragments detected in both *Msp*I and *Hpa*II profiles are unmethylated loci; Type II) fragments only detected in *Hpa*II profile are methylation of the external cytosine on only one strand (^HMe^CCG); Type III) fragments only detected in the *Msp*I profile are methylation of the inner cytosine on one or both strands (^HMe^CG or ^Me^CG); and Type IV) fragments absent from both *Hpa*II and *Msp*I profiles are methylation of both inner and outer cytosines on one or both strands (^HMe^C^HMe^CG, ^HMe^C^Me^CG, ^Me^C^HMe^CG, or ^Me^C^Me^CG), methylation of the both outer cytosine (^Me^CCG) and absence of restriction sites (Figure 2; Schulz et al., 2013). Multistate data matrices were generated by combining scores of the two parallel digestion profiles. To separate the contribution of unmethylated and methylated states of the same epiloci, the multistate epiloci were transformed into two separate binary subepiloci, u‐subepiloci (Figure 2; only type I scored as “1,” others scored as “0,” i.e., unmethylated epiloci) and m‐subepiloci (Figure 2; type II and type III scored as “1,” others scored as “0,” i.e., methylated epiloci). Thus, the multistate raw data matrices were recorded into two binary data sets: dataset U and dataset M following the “Mixed Scoring 1” approach (Figure 2; Schulz et al., 2013). This approach can temper the confusion of data interpretation between types II and III, which may be caused by the methylation variation at two closely spaced CCGG sites (Fulneček & Kovařík, 2014).

Intrapopulation methylation diversity was characterized by the percentage of private loci, percentage of polymorphic loci, and Shannon's information index for both u‐ and m‐subepiloci. Using GENALEX version 6.5 (Peakall & Smouse, 2006), population methylation differentiation (Φ_PT_) was determined based on both u‐ and m‐subepiloci for all intraspecific population pairs in both species by pairwise analysis of molecular variance (AMOVA) with 999 randomizations (Excoffier, Smouse, & Quattro, 1992). Pairwise distances between intraspecific individuals were computed by Euclidean measure for both u‐ and m‐subepiloci with GENALEX version 6.5. To visualize the methylation differentiation among populations of *C. robusta* and *C. intestinalis*, principal coordinates analyses (PCoA) of u‐ and m‐subepiloci were conducted separately with the individual‐by‐individual methylation distance matrices using GENALEX version 6.5.

As genetic analyses based on genomewide microsatellites have been conducted for *C. robusta* (Lin et al., 2017), we used *C. robusta* as an example to evaluate the relationship between genetic and methylation differentiation for populations using Genodive (Meirmans & Van Tienderen, 2004) with 99,999 permutations. The genetic differentiation matrix was obtained based on pairwise Φ_PT_ values calculated by 152 genomewide microsatellites from Lin et al. (2017), where the individuals used were exactly the same as these in this study. To investigate the relative contributions of genetic variation and environmental factors to the observed methylation variation, partial redundancy analysis (pRDA) was conducted for *C. robusta* using *vegan* package (function *varpart;* Oksanen et al., 2017) in the R (version 3.3.1.; R Core Team, 2016). The total methylation variation matrix, which combined U and M datasets, was set as the response matrix. The environmental factors and genetic variation matrices were set as explanatory variable matrices. Comparing with the annual mean temperature and salinity, recent studies emphasized that changes of maximum and minimum values may have greater effects on ascidians (Stachowicz, Terwin, Whitlatch, & Osman, 2002). Studies on both plants and animals suggest that the environmental stresses can be associated with long‐term epigenetic modifications, which can be transmitted through generations (Jablonka, 2012; Weyrich et al., 2016). Thus, the matrix of environmental factors was represented by the raw data of six environmental factors including annual water temperature, maximum water temperature, minimum water temperature, annual water salinity, maximum water salinity, and minimum water salinity. For the ambiguous invasion histories of both *C. robusta* and *C. intestinalis*, here we used the average values (among 1955–2012) of the six environmental factors collected from NOAA with the resolution as quarter‐degree (http://www.nodc.noaa.gov/OC5/SELECT/woaselect/woaselect.html). Although the environmental data from NOAA mainly focus on large‐scale variation in the open ocean, the difference of environmental factors among sample sites across continents still can be extracted from the NOAA datasets and is sufficient for our continent‐level study here. The genetic variation matrix was transformed from the 152 genomewide microsatellites data from Lin et al. (2017) according to the number of mutation steps among individuals (Leung, Breton, & Angers, 2016). To reduce the possible strong linear dependencies among the explanatory variables in pRDA model, forward selection method was applied on six environmental factors and microsatellites separately using R package *packfor* (function *forward.sel*; Dray, Legendre, & Blanchet, 2013), and the resulted variables were further used in pRDA. The total methylation variation explained by genetics or/and environmental factors was represented by the adjusted *R* ^2^ (*R*_adj_^2^), and permutation tests were performed to test the significance of each fraction with 999 randomizations (function *anova.cca* in package *vegan*; Oksanen et al., 2017).

To identify adaptive epiloci that likely are influenced by local environmental conditions, spatial analysis method (SAM) was performed for both *C. robusta* and *C. intestinalis* to test the relevance between allelic frequencies of epiloci and corresponding environmental parameters at sampling locations by computing multiple univariate logistic regression (Joost, Kalbermatten, & Bonin, 2008). Correlations between six environmental factors included in pRDA and each epiloci were conducted using SAM v.1 with the initial confidence level of 0.05. The input matrix included raw environmental data and methylated information for each individual. To ensure the robustness of SAM, two statistical tests including likelihood ratio G and Wald, which assess the significance of coefficients, were employed by the logistic regression function. A model was considered to be significant only if both tests reject the corresponding null hypothesis.

To verify whether these environment‐related epiloci were potentially involved in local environmental response, BAYESCAN (Foll & Gaggiotti, 2008) was used for both *C. robusta* and *C. intestinalis* to detect outliers by separating adaptive effects from neutral effects through the locus‐specific population differentiation coefficient. For each locus, BAYESCAN v2.1 directly calculates *q*‐values and an outlier is detected when its *q*‐value is lower than 5%. The input files (U and M datasets) were transformed as the format of dominant binary markers and tested following 10 pilot runs of 5,000 iterations with a 50,000 burn‐in and a thinning interval of 20. The prior odds for the neutral model were set as 10.

## 3 RESULTS

### 3.1 Intrapopulation methylation diversity

We generated 332 and 456 epiloci for *C. robusta* and *C. intestinalis*, respectively, by combining parallel results of *Eco*RI‐*Hpa*II and *Eco*RI‐*Msp*I reactions. Using the Mixed Scoring approach, 604 subepiloci including 275 u‐subepiloci and 329 m‐subepiloci for *C. robusta,* and 841 subepiloci including 395 u‐subepiloci and 446 m‐subepiloci for *C. intestinalis* were obtained by splitting the epiloci with more than one methylation type. The intrapopulation methylation diversity was measured by both u‐subepiloci and m‐subepiloci for *C. robusta* and *C. intestinalis* (Table 4). For both species, the percentages of private u‐subepiloci in each population were much higher than their corresponding values of private m‐subepiloci (Table 4). However, the polymorphism of u‐subepiloci, which ranged from 60.36% to 81.09% in *C. robusta* populations and varied from 67.59% to 73.92% in *C. intestinalis* populations (Table 4), were lower than corresponding estimators of m‐subepiloci in both *C. robusta* (79.03% to 92.40%; Table 4) and *C. intestinalis* (81.39% to 94.39%; Table 4). The values of Shannon index based on u‐subepiloci were similar among populations of *C. robusta*, ranging from 0.245 to 0.299 except for an European population BL (Blanes) where we detected a much higher value of 0.416 (Table 4). Interestingly, Shannon index of m‐subepiloci was much higher than that of u‐subepiloci in both *C. robusta* and *C. intestinalis* (Table 4). For both species, no obvious positive or negative correlations were detected between invasion time and methylation diversity at u‐ and m‐subepiloci. For *C. intestinalis*, one of native populations SC (Schleimünde) showed the highest values of polymorphism and Shannon index in both U and M profiles, while methylation diversity of the other native population SL (Salzhaff) was similar to that in invasive populations (Table 4).

**Table 4:** Methylation diversity for the analyzed populations of *Ciona robusta* and *C. intestinalis* based on u‐subepiloci (unmethylated loci) and m‐subepiloci (methylated loci)

Population ID	u‐subepiloci			m‐subepiloci		
	Private loci %	Polymorphic loci %	Shannon index	Private loci %	Polymorphic loci %	Shannon index
*C. robusta*						
AM (Arenys de Mar)	0.70	60.72	0.245	0	82.37	0.409
BL (Blanes)	3.27	81.09	0.416	0.30	82.67	0.433
SA (Cape town)	0.36	74.55	0.280	0	80.85	0.411
GAP (Gampo)	4.36	61.45	0.261	3.04	92.40	0.502
NMF (Nelson)	2.18	60.36	0.295	0	79.03	0.425
Average	2.17	67.63	0.299	0.67	83.46	0.436
*C. intestinalis*						
HF (Halifax)	0.25	73.67	0.290	0	91.70	0.385
MR (Murray River)	0.76	69.87	0.254	0	88.57	0.336
YM (Yarmouth)	0.25	70.38	0.279	0	90.36	0.356
SL (Salzhaff)	2.78	67.59	0.271	0	81.39	0.339
SC (Schleimünde)	2.53	73.92	0.295	0.2	94.39	0.496
Average	1.31	71.09	0.278	0.04	89.28	0.382

### 3.2 Interpopulation methylation differentiation

For *C. intestinalis*, higher Φ_PT_ values in U profile than those in M profile were observed in all population pairs (Table 5B). Putative native populations from Europe (SL and SC) were highly and significantly differentiated from invasive populations collected from North America (HF, MR and YM) in both U (Φ_PT_ = 0.133–0.216, *p *<* *0.05; Table 5B) and M profiles (Φ_PT_ = 0.053–0.083, *p *<* *0.05; Table 5B). The differentiation among three Canadian invasive populations (HF, MR & YM) was relatively low but significant in both U (Φ_PT_ = 0.018–0.069, *p *<* *0.05; Table 5B) and M profiles (Φ_PT_ = 0.010–0.023, *p *<* *0.05; Table 5B). The highest methylation differentiation was detected between two adjacent European native populations SC and SL in the U profile (Φ_PT_ = 0.224, *p *<* *0.05; Table 5B), while in M profile, the highest value was found between an European native population SL and a Canadian invasive population HF (Φ_PT_ = 0.083, *p *<* *0.05; Table 5B).

**Table 5:** Estimates of population methylation differentiation in two highly invasive ascidians *Ciona robusta* (A) and *C. intestinalis* (B). Above diagonal: pairwise Φ_PT_ based on m‐subepiloci, below diagonal: pairwise Φ_PT_ based on u‐subepiloci

(A)	AM	BL	SA	GAP	NMF
AM (Arenys de Mar)		0.058^a^	0.018^a^	0.132^a^	0.095^a^
BL (Blanes)	0.187^a^		0.067^a^	0.073^a^	0.037^a^
SA (Cape town)	0.047^a^	0.163^a^		0.124^a^	0.078^a^
GAP (Gampo)	0.274^a^	0.262^a^	0.220^a^		0.085^a^
NMF (Nelson)	0.262^a^	0.154^a^	0.184^a^	0.194^a^	

(B)	HF	MR	YM	SL	SC
HF (Halifax)		0.023^a^	0.010^a^	0.083^a^	0.058^a^
MR (Murray River)	0.054^a^		0.014^a^	0.077^a^	0.056^a^
YM (Yarmouth)	0.018^a^	0.069^a^		0.076^a^	0.053^a^
SL (Salzhaff)	0.210^a^	0.216^a^	0.215^a^		0.068^a^
SC (Schleimünde)	0.136^a^	0.156^a^	0.133^a^	0.224^a^	

a *p *< 0.01.

When interpopulation methylation differentiation was assessed by pairwise AMOVA, significant methylation differentiation in *C. robusta* was observed between all population pairs in both U and M profiles (*p *<* *0.05). Interestingly, overall pairwise Φ_PT_ values in U profiles were higher than their corresponding values in M profiles (Table 5A). The highest methylation differentiation for both U (Φ_PT_ = 0.274; Table 5A) and M profiles (Φ_PT_ = 0.132; Table 5A) was consistently observed between the population pair of AM (Arenys de Mar, Europe) and GAP (Gampo, Asia). The more recently introduced population GAP (Gampo) was also highly differentiated from the other three earlier introduced populations in both U (Φ_PT_ = 0.194–0.274; Table 5A) and M profiles (Φ_PT_ = 0.073–0.132; Table 5A). Intriguingly, the lowest Φ_PT_ values in both U (Φ_PT_ = 0.047; Table 5A) and M profiles (Φ_PT_ = 0.018; Table 5A) were detected between a European population AM (Arenys de Mar) and an African population SA (Cape Town), two populations with a distant geographical distance.

**Figure 3:**
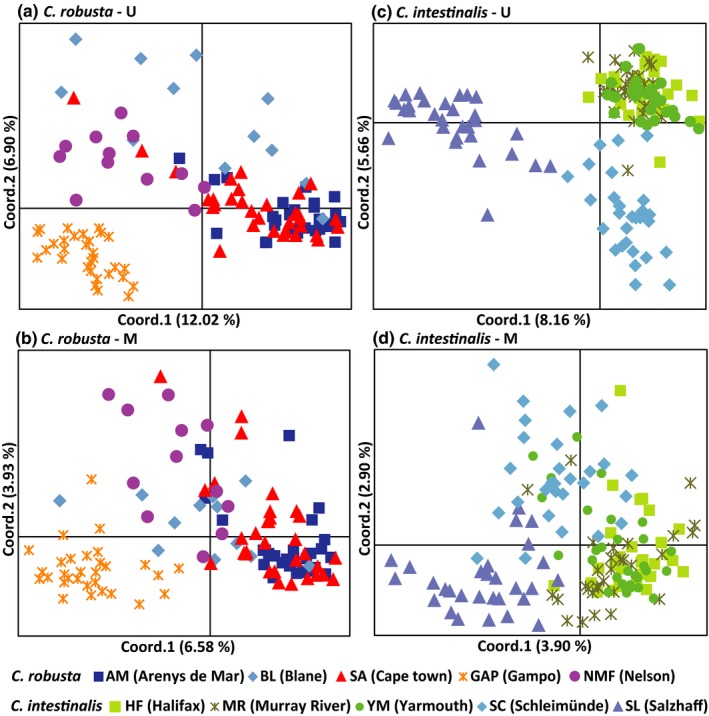
Principal coordinates analysis (PCoA) of U and M profiles for *Ciona robusta* and *C. intestinalis*. The first two coordinates are displayed with the indication of the percentage of variance explained in brackets. Populations collected from the same regions are represented by different shades of the same colors

Principal coordinates analysis (PCoA) of both U and M profiles showed largely consistent results with pairwise AMOVA for *C. robusta* and *C. intestinalis* (Figure 3). In the U profile of *C. robusta*, the Asian population (GAP) was completely separated from the other populations to form a unique cluster. Individuals of European population BL (Blanes) and Oceanian population NMF (Nelson) were scattered widely and slightly separated from the intermingled European (AM) and African (SA) populations (Figure 3a). In the U profile of *C. intestinalis*, PCoA of methylation distances separated introduced Canadian populations and putative native European populations. The three recently introduced Canadian populations, HF (Halifax), MR (Murray River), and YM (Yarmouth), were grouped together, while the two geographically close native populations from Europe (SL and SC) were completely distinguished from each other (Figure 3b). In the M profile, individuals were less clumped but clustered in a similar pattern as that of the U profile (Figure 3).

**Figure 4:**
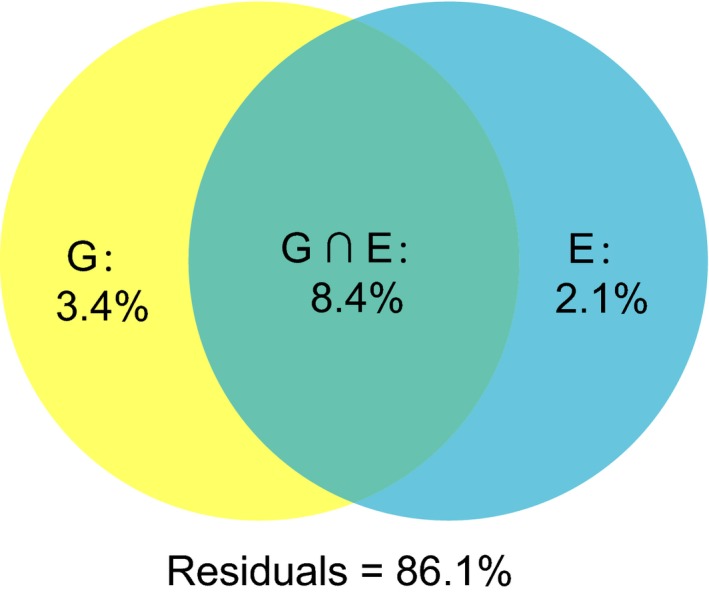
Results of variation partitioning analysis performed to assess the relative contribution of genetic variation and environmental effects to the total observed DNA methylation variation

Mantel tests showed significant correlation between genetic differentiation (Table 6) with methylation differentiation (Table 5A) in both U (*R *=* *0.700, *p *=* *0.017) and M (*R *=* *0.663, *p *=* *0.008) profiles, suggesting that genetic variation contributes to methylation variation observed in this study. In the pRDA mode, four environmental factors and 15 microsatellites were screened using the forward selection method for further analyses (Tables 7 and 8). The partitioning of total methylation variation showed that genetic variation and environmental factors could explain 11.8% (*p *=* *0.001) and 10.5% (*p *=* *0.001), respectively, of which 8.4% could be explained by both factors (Figure 4). Furthermore, 2.1% (*p *=* *0.001) and 3.4% (*p *=* *0.001) of the observed methylation variation can be solely explained by environmental factors and genetic variation, respectively (Figure 4).

**Table 6:** Estimates of population genetic differentiation (pairwise Φ_PT_) in *Ciona robusta* based on 152 microsatellites

	AM	BL	SA	GAP
AM				
BL	0.027*			
SA	0.030**	0.047*		
GAP	0.223**	0.193**	0.222**	
NMF	0.225**	0.172**	0.216**	0.106**

*p < 0.05; **p < 0.01.

**Table 7:** Screening results for environmental factors by the forward selection method

Variables	*R* ^2^	*R* ^2^ Cum	*R*_adj_^2^ Cum	*F*	*p*‐Value
MinS	0.060	0.060	0.052	7.256	0.001
MinT	0.027	0.087	0.071	3.304	0.001
MaxS	0.025	0.112	0.088	3.128	0.001
AveS	0.024	0.137	0.105	3.095	0.001

**Table 8:** Screening results for microsatellites by the forward selection method

Variables	*R* ^2^	*R* ^2^ Cum	*R*_adj_^2^ Cum	*F*	*p*‐Value
Cin27	0.064	0.064	0.056	7.767	0.001
Cin189	0.025	0.089	0.073	3.026	0.001
Cin104	0.018	0.107	0.083	2.300	0.001
Cin182	0.014	0.121	0.089	1.745	0.001
Cin211	0.011	0.133	0.093	1.426	0.002
Cin106	0.011	0.144	0.096	1.406	0.004
Cin141	0.011	0.155	0.099	1.363	0.001
Cin126	0.011	0.165	0.102	1.350	0.005
Cin72	0.010	0.176	0.105	1.321	0.013
Cin17	0.010	0.186	0.107	1.268	0.015
Cin162	0.010	0.196	0.110	1.280	0.017
Cin60	0.010	0.206	0.112	1.282	0.019
Cin76	0.010	0.215	0.115	1.281	0.012
Cin179	0.009	0.225	0.116	1.211	0.039
Cin160	0.009	0.234	0.118	1.203	0.041

### 3.3 Environment‐related subepiloci

**Figure 5:**
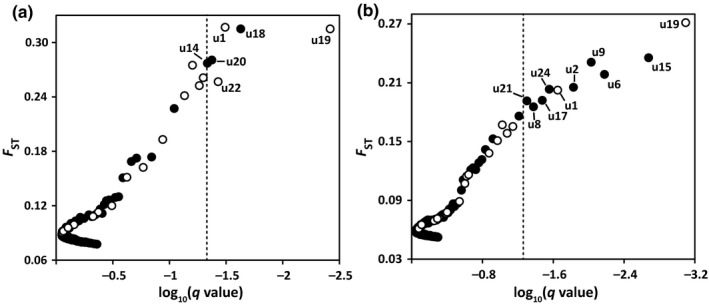
Epiloci under selection in *Ciona robusta* (a) and *C. intestinalis* (b) conducted by BAYESCAN. The dashed line donates the *q*‐value of 0.05. The open dots indicate that epiloci were correlated with environmental factors in the spatial analysis method (SAM)

**Figure 6:**
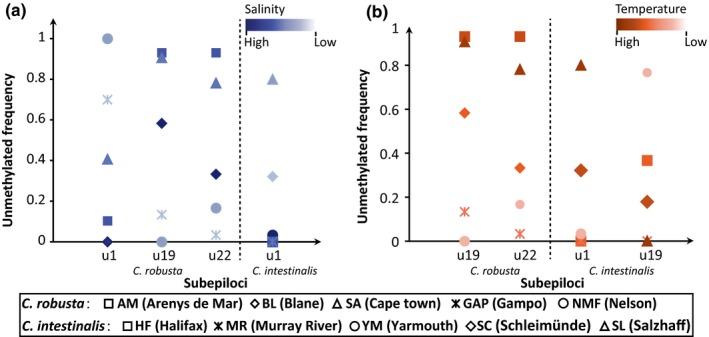
The unmethylation variation of u‐subepiloci identified by both the spatial analysis method (SAM) and BAYESCAN in different populations

SAM analyses detected that 24 (4.0%) and 20 (2.4%) subepiloci were correlated with at least one of the six environmental factors for *C. robusta* and *C. intestinalis*, respectively (Table 9). BAYESCAN identified six and 10 outliers for *C. robusta* and *C. intestinalis*, respectively (Figure 5; Table 9). Three environment‐related subepiloci in *C. robusta* and two in *C. intestinalis* were also identified as outliers in BAYESCAN (Figure 6; Table 9). The frequencies of environment‐related subepiloci varied along with the environmental factors and showed significant difference among populations (Figure 6). For *C. robusta*, seven subepiloci were temperature‐related, of which five were correlated with the minimum temperature. We detected 20 salinity‐related subepiloci in *C. robusta*, and 85% were related to the mean minimum salinity (Table 9). Furthermore, seven subepiloci were related to more than one salinity parameter, and three subepiloci were associated with both temperature‐ and salinity‐related parameters. For *C. intestinalis*, nine subepiloci were temperature‐related and 16 were correlated with salinity. Interestingly, 15 of 16 salinity‐related epiloci in *C. intestinalis* were correlated with the mean maximum salinity, mean minimum salinity, and average salinity at the same time, and five of nine temperature‐related subepiloci were also associated with salinity (Table 9).

**Table 9:** Epiloci detected by SAM and/or BAYESCAN

Epiloci	ID	MaxT‐related	MinT‐related	AveT‐related	MaxS‐related	MinS‐related	AveS‐related	BAYESCAN identified
*Ciona robusta*								
P1.157	u1				1	1	1	y
P1.227	u2					1		
P1.230	u3		1			1		
P1.286	u4				1		1	
P1.313	u5			1				
P1.341	u6					1		
P1.389	u7					1		
P2.163	u8					1		
P2.179	u9					1		
P2.197	u10					1		
P2.229	u11					1		
P2.240	u12					1		
P2.241	u13					1		
P2.308	u14							y
P2.322	u15				1		1	
P5.158	u16					1		
P5.296	u17				1		1	
P5.334	u18							y
P5.479	u19		1			1	1	y
P6.245	u20							y
P6.260	u21		1					
P6.306	u22		1			1	1	y
P6.313	u23				1	1	1	
P1.227	m1					1		
P1.389	m2					1		
P6.309	m3		1					
P6.367	m4	1						
*Ciona intestinalis*								
P1.200	u1			1	1	1	1	y
P1.445	u2							y
P2.177	u3				1	1	1	
P2.229	u4				1	1	1	
P2.265	u5	1						
P4.190	u6							y
P4.220	u7		1					
P4.312	u8							y
P4.362	u9							y
P4.382	u10			1	1	1	1	
P4.396	u11				1	1	1	
P5.189	u12				1	1	1	
P5.261	u13				1	1	1	
P5.300	u14			1	1	1	1	
P5.465	u15							y
P6.171	u16		1		1			
P6.209	u17							y
P6.214	u18	1		1				
P6.250	u19	1		1				y
P6.273	u20				1	1	1	
P6.298	u21							y
P6.388	u22			1	1	1	1	
P6.398	u23				1	1	1	
P6.406	u24							y
P1.198	m1				1	1	1	
P2.357	m2				1	1	1	
P2.410	m3				1	1	1	
P5.312	m4				1	1	1	

Note

Each locus is listed with ID, the association with temperature‐ and salinity‐related parameters, identified by BAYESCAN or not.

## 4 DISCUSSION

In this study, we explored the population methylation patterns of *C. robusta* and *C. intestinalis* at the genome level. Our results revealed highly consistent patterns in both invasive species. We detected a high degree of intrapopulation methylated polymorphism for both invasive species. In addition, both *C. robusta* and *C. intestinalis* showed significant population methylation differentiation and both genetic variation and varied local environments could partly explain the DNA methylation variation of *C. robusta*. We identified subepiloci presumably under selection and some of them were significantly correlated with environmental factors such as temperature and/or salinity. All results in our study suggest the putative contribution of DNA methylation variation to environmental response during biological invasions.

### 4.1 Methylation diversity within populations

For *C. robusta* and *C. intestinalis*, a great proportion of subepiloci was polymorphic, especially for m‐subepiloci (79.03%–92.40% in *C. robusta*, 81.39%–94.39% in *C. intestinalis*). The ratios of polymorphic loci were higher than those in other species, such as the tubeworm *F. enigmaticus* where only 50% of the methylation‐susceptible loci were polymorphic (Ardura et al., 2017). A study on a rare floodplain herb *Viola elatior* showed that the polymorphism among populations ranged from 22.7% to 61.8% for u‐subepiloci and varied from 30.6% to 61.8% for methylated subepiloci (Schulz, Eckstein, & Durka, 2014). Spontaneous epi‐mutations, which generally occur as the consequence of incorrect replication of the methylation pattern, may be one important reason for the high level of methylation polymorphism, as epi‐mutation rate can be several orders of magnitude higher than that of genetic mutations (Becker et al., 2011; van der Graaf et al., 2015; Hagmann et al., 2015; Schmitz et al., 2011). In addition, previous studies showed that *Ciona* ascidians are among the most genetically diverse animal investigated to date: The allelic polymorphism across the entire genome was 1.2% in *C. robusta* and the genomic average synonymous diversity was estimated to 0.05 per site in *C. intestinalis* (Dehal et al., 2002; Tsagkogeorga, Cahais, & Galtier, 2012). The high genetic variation can cause the absence of restriction enzyme sites, resulting in the high degree of methylated polymorphism.

The high level of intrapopulation methylation diversity can also be seen from the high values of Shannon index. Recent studies showed that environmental stresses could increase random methylation changes, leading to the high methylation divergence among individuals in the same populations (Huang et al., 2017; Morán et al., 2013; Richards et al., 2012). In a salt‐enriched experiment of brown trout (*Salmo trutta* L.), genomewide methylation changes were triggered after feeding on salt‐enriched diets for 2 days, and the largest methylation differentiation was observed at the fourth day after treatment (Morán et al., 2013). Interestingly, our recent study showed that *Ciona* ascidians could respond to rapid changing environments in the methylation profile, such as after 1 hr of high temperature exposure or after 3 hr of low salinity challenge, and environmental changes increased intrapopulation methylation variation (Huang et al., 2017). Many studies clearly showed that environment‐induced DNA methylation variation was heritable, thus such a type of DNA methylation variation can be relatively steadily maintained in populations (see the review by Verhoeven et al., 2016). Among *Ciona* populations collected from a broad geographical scale, multiple methylation features among populations, including the large number of population‐private epiloci, the high variation of polymorphic epiloci, and high values of Shannon index among populations, suggest that local environments may leave detectable methylation signatures at the whole genome level, particularly under the circumstance that *Ciona* species geographically spread less than one century ago (Zhan et al., 2015).

### 4.2 Remarkable methylation differentiation among populations

In our study, significant population methylation differentiation was found among populations for both *C. robusta* and *C. intestinalis*. The remarkable methylation differentiation can be influenced by various factors, including genetic background, spontaneous epi‐mutations, environmental induction and natural selection, and drift (Richards, 2006; Richards et al., 2017). For *C. robusta*, our results based on pRDA indicated that part of DNA methylation variation was explained by genetic variation. In addition to separate mechanisms shaping and maintaining genetic and DNA methylation variation, genetic and stable DNA methylation variation can be shaped simultaneously by the same local environmental pressures or neutral processes, leading to the correlation between genetic and methylation patterns (Preite et al., 2015). In the results of pRDA, more than 85% of the observed DNA methylation variation was not explained by available environmental factors and genetic variation, suggesting that the generation and/or maintenance of DNA methylation variation are complex processes and more variables including environmental and molecular ones may participate in such complex processes. More local environmental factors may influence DNA methylation variation at the population level, leading to a high degree of DNA methylation differentiation among populations collected from different environments. In addition, several recent studies suggest substantial genetic impacts on DNA methylation variation (Dubin et al., 2015; Roadmap Epigenetics Consortium et al., 2015). However, the contribution of genetic variation to DNA methylation variation was not as high as expected in this study. Two possible reasons may explain the observed patterns here: (a) genetic information based on 152 microsatellite markers was not enough to reflect the genomewide genetic variation among populations of *C. robusta*, and (b) two methods for both genetic and methylation analyses did not capture the same set of variation in the genome. These two possible reasons may result in poor correlation between detected genetic and DNA methylation variation in multiple analyses.

DNA methylation variation, at least part of it, can be independent from genetic variation in *C. robusta*. The direct evidence supporting this view comes from our pRDA analysis. In addition, the lowest population genetic differentiation was observed between two European populations AM (Arenys de Mar) and BL (Blanes), but the epigenetic pairwise Φ_PT_ value of this population pair was much higher than genetic differentiation estimators in both U and M profiles. Although a slight discrepancy of population genetic patterns can be detected when different markers are used to characterize genetic differentiation, the patterns of variation still appeared to be congruent among different types of markers (e.g., Maguire, Peakall, & Saenger, 2002). These two lines of evidence suggest that genetic component alone cannot explain the patterns observed in this study, and environmental factors played a role in shaping DNA methylation differentiation among populations.

The autonomous proportion of DNA methylation variation can be generated either randomly or environmentally induced, and both types can be environment‐related (van der Graaf et al., 2015; Hagmann et al., 2015). In a clonal fish *Chrosomus eosneogaeus*, random DNA methylation variation has been recorded in natural populations to cope with unpredictably changing environments (Leung et al., 2016). Studies on *Arabidopsis thaliana* showed that the stochastic changes in environment‐related methylation could be maintained over several generations and the accumulation of DNA methylation variance finally led to differentiation among populations (van der Graaf et al., 2015; Hagmann et al., 2015; Schmitz et al., 2011). Stress‐related methylation differentiation has been documented in experimental populations (Gao et al., 2010; Huang et al., 2017; Morán et al., 2013; Verhoeven et al., 2010). The stability of stress‐related DNA methylation variation varied from several hours to several generations (Herman, Spencer, Donohue, & Sultan, 2014; Huang et al., 2017). If the same advantageous phenotype arose every generation repeatedly by transient methylated modifications, such changes are expected to contribute to the interpopulation methylation differentiation in different environments (Schulz et al., 2014). The environment‐related DNA methylation variation may have an effect to maximize individual fitness to local ecological conditions. Studies of three acroporid corals showed that DNA methylation variation may influence their tolerance to the thermal stress and ocean acidification (Dimond & Roberts, 2016). In wild baboons *Papio cynocephalus*, a large number of differentially methylated regions were found near metabolism‐related genes, suggesting the epigenetic regulation for catering to different resource availability (Lea et al., 2016). For invasive species, recent works showed the correlation between methylation differentiation and diverse phenotypes in different invaded habitats, suggesting the potential role of DNA methylation variation in shaping different phenotypes to respond to varied environmental stresses (Gao et al., 2010; Richards et al., 2012). Despite that only a small proportion of DNA methylation variation was explained solely by collected environmental factors, fitness effects of some loci may be sufficient to make *Ciona* populations adapt to local environments. Our previous study also showed that a limited number of CpG loci and/or genes were involved in environmental adaptation (Huang et al., 2017; Pu & Zhan, 2017).

### 4.3 Environment‐related epiloci

We detected that subepiloci of *C. robusta* (4.0%) and *C. intestinalis* (2.4%) significantly correlated with changes of environmental factors by SAM. Similarly, 14 epiloci (4.5%) were identified to significantly correlate with ecoclimatic variables in the study of Orchids *Dactylorhiza* (Paun et al., 2010). However, the proportion of environmental‐related subepiloci in our study was much less than that in red grouse *Lagopus lagopus scotica* associated with gastrointestinal parasite load (13.6%; Wenzel & Piertney, 2014). Likewise, 12% of polymorphic epiloci in *Spartina alterniflora* were found to be correlated with oil exposure (Robertson, Schrey, Shayter, Moss, & Richards, 2017). The different ratios among studies may result from the difference of stresses and degree of stresses. In concert, findings in related studies indicated that environmental factors modulated the establishment and maintenance of methylation modifications to specific stress‐related genes (Paun et al., 2010; Pu & Zhan, 2017). Although epiloci related with temperature and salinity in *C. robusta* and *C. intestinalis* were identified, it was still unknown whether these environment‐related epiloci were identical in certain groups responding to temperature or salinity in this study. This comparison cannot be clarified by using fragments acquired from MSAP, because fragments from different species with the same sizes may represent different sequences (Caballero et al., 2008), even they are the products of the same selective primers.

In this study, subepiloci with significant methylation differentiation among populations were identified by BAYESCAN, which may be involved in local adaptation (Foll & Gaggiotti, 2008). The overlap at subepiloci of SAM with BAYESCAN suggests that these environment‐related subepiloci may contribute to the divergence of methylation pattern among populations. This hypothesis was confirmed by another specific study in populations of *C. robusta*, where we detected the correlation between methylation patterns of key genes and environmental factors (Pu & Zhan, 2017). Significant correlation was observed between methylation levels and water temperature at several CpG sites in heat shock protein 90 (HSP90), while one CpG site in Na^+^‐K^+^‐2Cl^−^ cotransporter (NKCC) showed significant association with salinity (Pu & Zhan, 2017). As the technique of MSAP can only produce anonymous fragments with unknown genetic contexts, we cannot identify whether the outliers detected in these two studies play a consistent role in the process of response to environmental changes. All these results demonstrated that varied local environments may have important influence on the methylation patterns in populations. Therefore, we infer that DNA methylation may enhance invaders’ adaptive capacity to different habitats by extending the flexibility of a genotype to respond differentially under variable environmental conditions.

### 4.4 Technical issues

MSAP is a method based on PCR amplification of selected restriction fragments, which can be affected by both methylation modifications and genetic mutations of restriction sites. Therefore, MSAP data matrices inevitably include part of genetic information. The ambiguous definition of type IV fragments in MSAP makes it difficult to distinguish between genetic and methylation variation. Although the polymorphism level of epiloci and population methylation differentiation may be affected by the mixed genetic variation, the role of local environmental factors in shaping DNA methylation variation can still be inferred from results of pRDA and SAM analyses. In order to reduce the confusion in data interpretation between type II and type III, the “Mixing Scoring 1” approach was used in our study to combine both type II (HPA+/MSP−) and type III (HPA−/MSP+) as methylated epiloci. The use of this strategy resulted in more conservative results but some degree of loss of methylation variation among individuals. As MSAP has its technical disadvantages (Schulz et al., 2013), whole epigenome sequencing‐based methods are needed in future studies to obtain more accurate epigenetic information.

## AUTHOR CONTRIBUTIONS

A.Z. conceived the study; P.N. and A.Z. designed the experiment. P.N. conducted the experiments and analyzed the data; S.L., Y.L., W.X., and X.H. did both laboratory and field works; P.N. and A.Z. wrote the manuscript, and all authors contributed to the revisions; all authors reviewed and commented on the manuscript.

## DATA ACCESSIBILITY

All raw MSAP data and transformed MSAP data for both species, and raw SSRs data for *C. robusta* used in this study have been deposited into Dryad (https://doi.org/10.5061/dryad.q8v97).
